# The hypothalamic-pituitary-adrenal axis and the central monoaminergic systems: a pathophysiological link to insomnia with clinical implications

**DOI:** 10.5935/1984-0063.20220032

**Published:** 2022

**Authors:** Marcio Luciano de Souza Bezerra, Raimundo Nonato Delgado Rodrigues, Ricardo Oliveira Souza

**Affiliations:** 1 RioSono Sleep Center, Sleep Medicine - Rio de Janeiro - RJ - Brazil.; 2 Unidade do Sono de Brasilia DF, Sleep Medicine - Brasilia - DF - Brazil.; 3 Instituto de Pesquisa da Rede D’Or, Neurology - Rio de Janeiro - RJ - Brazil.

**Keywords:** Autonomic Nervous System, Hypothalamic Hormones, Adrenal Cortex Hormones, Sleep, Sleep Deprivation

## Abstract

The authors present a theoretical overview of the hypothalamic-pituitary-adrenal axis and the central monoaminergic systems, focusing on a putative pathophysiological relationship to insomnia complaints. Insomnia is an independent and self-perpetuating disorder that requires diagnostic and therapeutic attention in the presence of concomitant disorders, likely with bidirectional influence. An adequate understanding of such mechanisms can help for a better understanding of the interplay between clinical features, neurotransmitters, and the adrenal hypothalamic-pituitary axis may help clinicians to better manage insomnia patients

## INTRODUCTION

Sleep disorders galvanize the attention of physicians as well as laypeople as a relevant risk for common physical and neuropsychiatric disorders. Indeed, insomnia, one of the most prevalent among the sleep disorders, has been notoriously linked to a variety of health problems^1,2^.

Historical references to insomnia date back to the dawn of civilization^3^. The scientific investigation on its etiology and pathogenesis has nevertheless evolved quite slowly. Several factors may be accounted for, including the heterogeneity of insomnia symptoms, the lack of objectively defined phenotypes, and the discrepancy between self-reports and objective measures of sleep using polysomnography.

The term insomnia may refer both to a symptom, as well as to a host of disorders, which have been defined in various diagnostic systems. For the present essay, we used two classification systems: “International Classification of Sleep Disorders - 3rd edition (ICSD-3)”^4^ and the fifth edition of the “Diagnostic and Statistical Manual of Mental Disorders (DSM-5)”^5^. Both define insomnia operationally as frequent and persistent difficulty in initiating or maintaining sleep, or morning awakening despite adequate opportunity for sleep. Furthermore, the disorder must lead to significant impairment of daily living activities and is not better explained by other mental or physical illnesses.

Insomnia is commonly conceptualized within a diathesis-stress framework in which it is thought to result from predisposing (vulnerability or protective traits), precipitating (initiating events that trigger insomnia), and perpetuating (behaviors and cognitions that contribute to the maintenance of insomnia) factors^6^.

Sleep is inserted in a complex network of interconnected brain regions involving several neurotransmitters and neuromodulators. Among them, the monoaminergic pathways that seem to be responsible for the modulation of the sleep/wake cycle. The interruption and/or demodulation of the monoaminergic system may promote certain diseases. Menon et al. (2019)^7^ reported, during sleep deprivation (SD), that monoamine levels remained high even during the subsequent recovery.

At the beginning of stage N2, the activity of the HPA axis (corticotropin-cortisol releasing hormone) decreases progressively, reaching its nadir during slow-wave sleep. By the same token, plasma cortisol levels are also diminished during most of REM sleep time, possibly due to a general inhibition of peripheral and muscular autonomic activity^8^. The elevation of adrenocorticotropic hormone (ACTH) in the morning is one of the factors that contribute to the end of the hypnic period^9^. Insomnia patients with objectively fragmented nighttime sleep show elevated 24-h plasma ACTH and cortisol levels^10^ mostly in the evening and the first half of the night^1^. Chronic activation of the hypothalamic-pituitary-adrenal axis suggests that insomniacs are at risk not only for mental disorders but also for significant medical comorbidities^6^.

Indeed meta-analyses demonstrate that insomnia is a major risk factor for depression^11^, cardiovascular diseases^12^, hypertension^13^, and diabetes^14^. Furthermore, insomnia has been shown to negatively impact the quality of life^15^, daytime functioning, and disability-adjusted life years^16^. Thus, early identification and reduction of risk factors are important parts of health maintenance.

It is postulated that: i) a balanced synthesis of monoamines in the central nervous system is a prerequisite for normal brain function; ii) the hypothalamic-pituitary-adrenal (HPA) axis is an adaptive system to maintain a dynamic equilibrium or homeostasis in a constantly changing environment; iii) these former two postulates may also apply to several other pathological conditions (sleep- related or not), that might guard with insomnia disorders a traceable relationship.

To further evaluate the postulates above, we will study the interplay between HPA, biogenic amines, and clinical disorders within and outside the field of sleep medicine, as follows:

### 1. Biogenic amines in clinical disorders:

#### 1.1. Serotonergic system

##### 1.1.1. Migraine

Migraine affects 11% of the adult population worldwide^17^. The National Health Insurance Service of South Korea reported that the prevalence of migraine has gradually increased annually^18^. Kim et al. (2018)^18^ found that insomnia is a greater risk factor for migraine than other types of sleep disorder. Lin et al. (2016)^19^ found higher migraine frequency correlates with poorer sleep quality, as indicated by a higher Pittsburgh sleep quality index (PSQI). The occurrence of migraine attacks has been associated with high stress and inadequate sleep in the 2 days before an attack^20^. Sleep deprivation can exacerbate self-reported pain^15^. This exacerbation may involve impaired descending pain inhibitory functioning and deficiency of serotonin-related descending pain inhibition^16,17^. On the other hand, sleep has always been known to be an efficient means to abort migraine attacks^21^.

Serotonin vasoconstriction of blood vessels at the nerve endings influences nociceptive pain^22^. Comings et al. (1994)^23^ postulated that low serotonin levels dilate blood vessels and trigger migraine. The efficacy of triptans in migraine probably lay on their affinity to the serotonin receptors of the trigeminal nerve and cranial vessels^17^.

Symptoms of autonomic dysfunction are common in patients with migraine, both during and between migraine attacks. Results are conflicting on the role of the sympathetic versus parasympathetic dysfunction seen in this disorder. Most studies reported reduced sympathetic function^24^.

Patacchioli et al. (2006)^25^ recorded cortisol levels in the saliva of patients with a chronic migraine that were consistently higher compared to healthy individuals.

##### 1.1.2. Depression

Depression is a mental disorder associated with significant morbidity and mortality^26^. Episodes of major depression and insomnia commonly co-occur and non-depressed people with insomnia have a twofold risk of developing depression compared to people who sleep well^6^. Fernandes-Mendoza et al. (2015)^27^ found that insomnia with objectively documented short sleep duration is associated with incident depression regardless of accessory psychological factors such as coping resources.

Although the early idea that a single neurochemical is the cause of depression is now considered simplistic, the low serotonin hypothesis still lies at the foundation of most research on depression^28^. Despite decades of research, the role serotonin plays in depressive phenotypes has not been conclusively determined. Andrews et al. (2015)^29^ propose that altered serotonin transmission is part of the evolved process by which depression is regulated because they have reviewed a large body of evidence indicating that the opposite appears to be true. For the depressive phenotypes — sickness behavior, starvation depression, and melancholia — serotonin transmission to multiple brain regions appear to be elevated.

One of the most consistent findings in the biology of depression is altered activity of the (HPA) axis. A large body of evidence indicates that its activity is significantly heightened in patients suffering from depression, compared with healthy controls^30,31^.

Sheena et al. (2020)^32^ demonstrate that depressed patients present heart rate variability and autonomic balance in favor of an increased sympathetic tone. A high sympathetic and/or low cardiovagal activity in major depression may contribute to the higher cardiac morbidity and mortality among these patients.

##### 1.1.3. Attention-deficit-hyperactivity-disorder (ADHD)

ADHD affects around 5% of children and 3% of adults’ worldwide^33^. Both ADHD and insomnia are heterogeneous disorders, and the relationship between them is complex. Cross-sectional clinical and population studies have shown that insomnia is associated with adult ADHD in 43-80%^34^ regardless of the pharmacological treatment for ADHD^35^. Neuroanatomical, pharmacological, and genetic studies have provided evidence for the involvement of serotonergic orbitofrontal-striatal circuits in discrete dimensions of ADHD^36^, especially in hyperactivity and impulsivity, but perhaps not in inattention^37^.

Meta-analysis studies have found evidence of autonomic nervous system dysfunction in ADHD, either in the direction of hypo or hyperexcitation. Indeed, most of the significant differences between the groups were due to hypoactivation of the autonomic nervous system^32^.

Isaksson et al. (2012)^38^ verified the levels of salivary cortisol of patients with ADHD. The main findings of this study were that children with ADHD had significantly lower levels of cortisol in the morning and evening than the unaffected individuals. This association between a down-regulated HPA axis and ADHD seems to corroborate the theories that imply ADHD as a consequence of hypo-arousal.

#### 1.2. Dopaminergic system

##### 1.2.1. Parkinson’s disease (PD)

Parkinson’s disease (PD) is the second most common neurodegenerative disorder. Poor sleep and excessive alertness affect as many as 90% of patients and stand among the commonest non-motor symptoms of PD^39^. Sleep maintenance insomnia occurs in up to 50% of PD patients^40^. The pathophysiology of sleep-wake disturbances in PD remains largely unknown, but the etiology is likely to be multifactorial. Insomnia is the most prevalent sleep disorder in PD, while sleep fragmentation and early awakening being the commonest phenotypic manifestations^27^. Biochemical changes in monoaminergic and cholinergic systems involved in sleep regulation are potential causative factors for sleep interruption in PD. A decline in the number of serotonergic neurons in the dorsal raphe nuclei, noradrenergic neurons in the *locus coeruleus*, as well as cholinergic neurons in the pedunculopontine nucleus, have been described^41^. Besides, degeneration of the ventral tegmental area, which appears to be involved in the regulation of sleep-wake via dopamine D1 receptors, may have a substantial impact on sleep disorders in PD^42,43^.

Autonomic dysfunction is an essential category of non-motor phenotypes in PD, which includes gastrointestinal dysfunction, cardiovascular dysregulation, urinary disorder, sexual dysfunction, thermoregulatory aberration, and pupil-motor and lacrimal abnormalities. The distribution and frequency of dysautonomic symptoms in patients with PD depend on the stage of the synucleinopathic degeneration and the site of involvement^44^.

Plasma ACTH levels in patients with untreated idiopathic PD are significantly low compared to healthy controls^45^. PD related dopaminergic deficits are likely to disrupt normal pituitary function and ACTH secretion^46^.

##### 1.2.2. Restless legs syndrome (Willis-Ekbom disease)

Restless legs syndrome (RLS) is a disorder characterized by an intense and irresistible urge to move the legs, usually associated with sensory complaints and motor restlessness. The symptoms worsen at rest, increase in severity in the evening or at night, and are relieved by movements^47^. Restless legs syndrome is a common sensorimotor disorder with a prevalence of up to 11% in the general population and is more frequent in females than in males^48^. Insomnia is the commonest reason for a patient with RLS to search for care in clinical practice. The most common bedtime problem caused by RLS is difficulty-initiating sleep^49^. A circadian pattern can be found that worsens the urge to move the legs in the evening, alleviating in the morning after waking up^50^.

A large number of clinical and pharmacological studies have provided evidence for the role of dopaminergic dysfunction in RLS^51^. Dopaminergic A11 cells, located in the midbrain and close to the hypothalamus are the major source of dopamine in the spinal cord. A11 cells reach the dorsal horn and then project to the motoneuronal pool^52^. Stereotactic bilateral 6-hydroxydopamine lesions of the A11 nucleus increase the number of standing episodes and the total standing time compared to the sham-operated rats^53^, suggesting an important role of A11 dopaminergic cells in pathophysiological pathways in RLS.

In the literature, the results of the cortisol level in RLS are conflicting. Schilling et al. (2010)^54^ found significantly increased nocturnal cortisol excretion in RLS, demonstrating nocturnal hyperactivity of the HPA system in RLS. Notwithstanding, Lattova et al. (2011)^55^, found no abnormalities in the regulation of the HPA system in patients with RLS. In another study, ACTH levels were slightly lower compared to controls, while cortisol levels were similar between groups^50^.

Izzi et al. (2014)^56^ evaluated patients with RLS who tended to develop hypertension, with a reduced range of sympathetic and parasympathetic responses in the head tilt test. These findings may support the hypothesis of an involvement of the autonomic nervous system in RLS and, consequently, an increased cardiovascular risk^56^.

#### 1.3. Histaminergic system

##### 1.3.1. Allergic rhinitis

Histamine plays a critical role in the symptoms of allergic rhinitis; it is present in nasal mast cells and circulating basophils and is released following allergen challenge. Histamine is released during the initial and final phases of the nasal allergic response^57^.

Buske-Kirschbaum et al. (2010)^58^ reveal data on attenuated HPA axis responsiveness to stress in atopic conditions and suggest that HPA axis hypo- responsiveness in atopy may be linked to the severity of the allergic inflammatory process^58^.

In the study conducted by Ishman et al. (2007)^59^, specific sympathetic hypofunction was identified in all patients with allergic rhinitis.

By the same token, using multivariable logistic regression, Leger et al. (2017)^60^ found that patients suffering from severe persistent allergic rhinitis were more likely to experience difficulty falling asleep, nocturnal awakenings, insomnia, and poor-sleep than those with other types of allergic rhinitis.

##### 1.3.2. Skin disorders

Atopic dermatitis (ATD), one of the most common chronic allergic skin inflammatory diseases, has an increasing prevalence in the population, affecting up to 20% of children and 10% of adults in high-income countries^61^. Current evidence suggests that the pathogenesis of ATD is multifactorial, (epidermal barrier dysfunction, inflammation induced by T-cell type 2 (Th2) lymphocytes), but is also modulated by neuroendocrine mediators, such as histamine via the histamine H4 receptor^62^.

The skin, the largest organ in the body, has developed a system to keep itself healthy in the face of environmental, biological, and emotional stressors. With that, we have a peripheral HPA axis similar to the central HPA axis^63^. There are complex interactions between the central and cutaneous axes that have their effects on the skin barrier and dermatitis. The HPA peripheral axis shows a hierarchical structure similar to the central axis. Studies show that HPA dysfunction is present in patients with ATD due to the decreased response of elevated glucocorticoids to stress compared to those not affected by ATD^62^.

Mostaghimi and Hetze (2019)^64^ described patients with inflammatory skin disorders (psoriasis and chronic eczema) with a greater chance of insomnia compared to patients with non-inflammatory skin cancer. Sleep is disturbed in 60% of children with atopic dermatitis. Using actigraphy and sleep diaries in children with atopic dermatitis, Fishbein et al. (2018)^65^ found that the time awake after sleep onset was longer and sleep efficiency was lower. Their parents reported that they were significantly more restless during sleep and had more daytime sleepiness compared to controls.

#### 1.4. Adrenergic system

##### 1.4.1. Autism spectrum disorder (ASD)

Autism refers to a broad range of conditions characterized by challenges with social skills, repetitive behaviors, speech, and non-verbal communication. About 1 in 54 children has been identified with autism spectrum disorder (ASD) according to estimates from CDC’s autism and developmental disabilities monitoring^66^.

Lake et al. (1977)^67^ have demonstrated findings suggestive of increased noradrenergic activity in ASD, including increased plasma epinephrine and norepinephrine.

Symptoms of insomnia are frequently reported for individuals with an autism spectrum disorder, though the underlying causes of these sleep problems remain unclear. In children and adolescents with ASD, sleep problems often persist into adulthood^68^.

Richdale and Prior (1992)^69^ observed a trend of hypersecretion of daytime cortisol in 19 children with ASD, with this effect being more pronounced in older children (>8 years). Other studies have found that children with ASD had consistently high nighttime cortisol levels and more variable cortisol levels compared to control children^70^. Baker et al. (2019)^71^ observed that patients with unmedicated ASD had significantly greater reductions in nighttime cortisol concentrations compared with ASD participants medicated for depression and anxiety. Moreover, the low cortisol levels observed in ASD- only adults suggest dysregulation of the hypothalamic-pituitary-adrenal (HPA) axis^71^.

Growing evidence has shown the association of ASD with the dysfunction of the autonomic nervous system (ANS)^72^. A wide range of studies with variable samples and methodologies have revealed the overall boosted sympathetic nerve system and decreased vagal tone in autistic children^73^.

### 2. HPA and clinical disorders

#### 2.1. Cardiovascular diseases

Out of the multiple mechanisms underlying the relationship between cardiovascular disease and insomnia, the dysregulation of the hypothalamic- pituitary axis holds a central position^74^. There is evidence that both the adrenocorticotropic hormone and cortisol secretions are increased in patients with insomnia, particularly those with objective short sleep duration, suggesting increased HPA axis activity^1,75^. The Sleep Heart Health Study conducted a cross-sectional analysis of sleep duration (measured by subjective report and polysomnography) and prevalence of hypertension. Participants with a subjective report of fewer than 7 hours of sleep per night had a higher odds ratio for hypertension^76^. Short sleep duration is also associated with increased coronary heart disease (CHD) mortality and morbidity^77^. Meanwhile, Chandola et al. (2010)^78^ found that sleep duration of fewer than 6 hours also shows a significantly increased risk for CHD when combined with insomnia but not when looked at alone.

#### 2.2. Cerebrovascular disease

Insomnia incidence in patients who have had a stroke approaches 57%, with 38% reporting insomnia as a preceding symptom to the stroke^79^. Leng et al. (2015)^80^ examined the association between self-reported short-sleep duration and risk of stroke, depict that sleep duration less than five to six hours was associated with a 15% increased risk of incident stroke compared to six to eight hours with no evidence for heterogeneity across studies. Helbig et al. (2015)^81^ followed 17,604 subjects aged 25 to 74 for a mean of 14 years and found that sleep duration less or equal five hours compared to seven to eight hours was associated with a trend toward increased risk of stroke in men but not women^81^.

Barugh et al. (2014)^82^ conducted a systematic review to assess cortisol levels in acute stroke and their outcome. Elevated cortisol levels were correlated with increased stroke severity in most studies that explored this association. Cortisol levels are elevated for at least 7 days after the stroke and within the normal range for most people within 3 months.

#### 2.3. Obesity

The National Health and Nutrition Examination Survey reported that of obesity was 42.4% among U.S. adults in 2017-2018^83^. In obese individuals, the HPA axis is altered and cortisol concentrations are elevated in adipose tissue due to elevated local activity of type 11β-hydroxysteroid dehydrogenase^84^. Pasquali et al. (1993)^85^ examined two groups of obese women, who were divided into a group with abdominal obesity and another with peripheral obesity. The rates of urinary excretion of free cortisol were significantly higher in individuals with abdominal body fat distribution. These results suggest that these obese women may have hypothalamic-pituitary-adrenal hyperactivity^85^.

Rasmussen et al. (2008)^86^ found a significant decrease in total sleep duration in obese individuals, a reduction of 88 minutes, compared with non-obese individuals. A significant decrease in total sleep duration was still present in obese individuals after weight loss compared to non-obese individuals. The duration of REM and non-REM periods was similar in obese and non-obese individuals.

An increase in autonomic nervous system activity has been demonstrated in obese patients, particularly in the muscular and renal vasculature^87^. Landsberg et al. (2001)^88^ confirmed the hypothesis of hyperactivity of the autonomic nervous system in obese individuals, with or without hypertension. Orexins A and B are important homeostatic mediators of central control of energy metabolism and maintenance of sleep/wake states.

The dysregulation or loss of orexin signaling has been associated with sleep disorders and obesity. As a multifaceted peptide, orexin is likely to play an integrative role, coordinating the central modulation of sleep and physical activity in the context of energy balance. Evidence suggests that milder disorders of the orexin system can disrupt normal orexinergic blocking of sleep/wake state transitions and thus contribute to poor quality sleep^89^.

## CONCLUSION

At this point one may endeavor to inquire which could be the practical conclusion of all the above mentioned.

The goal of the present essay is to provide a selective review of the interplay between insomnia and activation of the stress system (hypothalamic-pituitary-adrenal axis and the sympathetic system), and the role of a heightened release/turnover of brain monoamines. These neurotransmitters actions may reproduce the common clinical phenotypes of insomnia. So, according to our rationale, different patterns of dysfunction in these neurotransmitter systems can be determine different types of insomnia.

It is known that, among vertebrates, to induce various physiological and behavioral changes, the monoaminergic systems and the hypothalamic-pituitary- suprarenal axis are activated and adaptation to extrinsic and intrinsic forces is a survival necessity for all living organisms^90^. The brain’s premise is to enable the organism to cope with stress and its inherent unpredictability, transforming routine into a habit, because without having excessive use of neurotransmitters, it manages to save more energy. The brain needs to reframe chronic stimuli to protect us from the damage it has suffered.

Stress accelerates release, turnover, promotes fluctuations in the concentrations of these neurotransmitters, and activates HPA axis function. Under acute stress, transient changes in those systems have to occur in order to restore homeostasis; besides, chronic stress accompanied by repetitive and/or prolonged stimulation is prone to induce a long-lasting imbalance in central monoaminergic systems to neurons^88^, as well as in the HPA axis, that may manifest not only as a general decline in physical conditioning and health, but also in neurological and immune function (especially during periods of growing and development)^91^. Thus, human organism needs to react appropriately to the demands of the internal and external environments, and that is why neural networks are called upon to reorganize themselves, to provide the necessary substrates for homeostasis. However, the chronic stimulation of these systems, in susceptible individuals, can also produce clinical conditions simultaneously.

**Figure 1:**
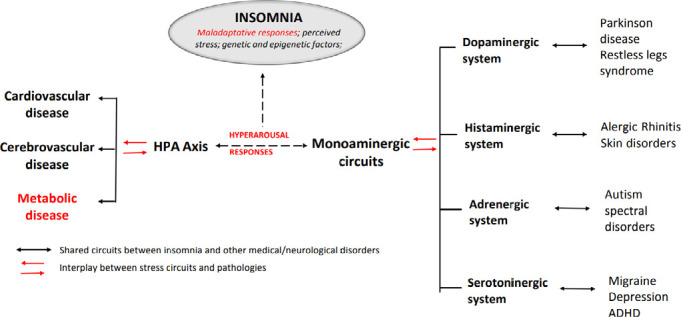
Possible physiopathological interplay between insomnia disorders and neurotransmitter circuits. See text for details.

**Notes:** ADHD = Attention deficit and hyperactivity disorder; HPA = Hypothalamic-pituitary axis.

Finally, we believe that the recognition and understanding of the systems involved and their interactions can provide, valuable information for the recognition and management of the specific clinical phenotypes of insomnia (see Figure 1 for summary).
